# Water availability, bedrock, disturbance by herbivores, and climate determine plant diversity in South-African savanna

**DOI:** 10.1038/s41598-021-02870-3

**Published:** 2022-01-10

**Authors:** Martin Hejda, Jan Čuda, Klára Pyšková, Guin Zambatis, Llewellyn C. Foxcroft, Sandra MacFadyen, David Storch, Robert Tropek, Petr Pyšek

**Affiliations:** 1 Institute of Botany, Department of Invasion Ecology, Czech Academy of Sciences, 25243 Průhonice, Czech Republic; 2 Department of Ecology, Faculty of Science, Charles University, Viničná 7, 12844 Prague, Czech Republic; 3 Scientific Services, South African National Parks, Private Bag X402, Skukuza, 1350 South Africa; 4 Centre for Invasion Biology, Department of Botany and Zoology, Stellenbosch University, Stellenbosch, South Africa; 5 Department of Mathematical Sciences, Stellenbosch University, Stellenbosch, South Africa; 6 Center for Theoretical Study, Charles University, Prague, Czech Republic; 7 Biology Centre, Institute of Entomology, Czech Academy of Sciences, Branišovská 31, 37005 České Budějovice, Czech Republic

**Keywords:** Community ecology, Conservation biology, Ecosystem ecology, Plant sciences

## Abstract

To identify factors that drive plant species richness in South-African savanna and explore their relative importance, we sampled plant communities across habitats differing in water availability, disturbance, and bedrock, using the Kruger National Park as a model system. We made plant inventories in 60 plots of 50 × 50 m, located in three distinct habitats: (i) at perennial rivers, (ii) at seasonal rivers with water available only during the rainy season, and (iii) on crests, at least ~ 5 km away from any water source. We predicted that large herbivores would utilise seasonal rivers’ habitats less intensely than those along perennial rivers where water is available throughout the year, including dry periods. Plots on granite harboured more herbaceous and shrub species than plots on basalt. The dry crests were poorer in herb species than both seasonal and perennial rivers. Seasonal rivers harboured the highest numbers of shrub species, in accordance with the prediction of the highest species richness at relatively low levels of disturbance and low stress from the lack of water. The crests, exposed to relatively low pressure from grazing but stressed by the lack of water, are important from the conservation perspective because they harbour typical, sometimes rare savanna species, and so are seasonal rivers whose shrub richness is stimulated and maintained by the combination of moderate disturbance imposed by herbivores and position in the middle of the water availability gradient. To capture the complexity of determinants of species richness in KNP, we complemented the analysis of the above local factors by exploring large-scale factors related to climate, vegetation productivity, the character of dominant vegetation, and landscape features. The strongest factor was temperature; areas with the highest temperatures reveal lower species richness. Our results also suggest that *Colophospermum mopane*, a dominant woody species in the north of KNP is not the ultimate cause of the lower plant diversity in this part of the park.

## Introduction

In the era of Anthropocene, a considerable amount of Earth’s biological diversity is concentrated in protected areas^1^. The maintenance of biodiversity within these areas depends on careful management, enabling crucial ecosystem processes to operate sustainably, allowing for an appropriate response to current global change. This is especially relevant in African savannas, a major biome, which is threatened by large-scale habitat conversion and whose biota depend on intricate relationships between plants and animals^2^. South-African savannas rank among biomes with a high conservation value; the enormous diversity of various groups, including vascular plants, birds, mammals, and insects, depends on the iconic large populations of megaherbivores, acting as keystone species^3^.

Savanna plant diversity and structure is affected by four key determinants: soil moisture, soil nutrients, herbivory, and fire^4–10^, and the combined effects of all these factors result in spatially heterogeneous patterns of plant community composition and diversity^5,11,12^.

(i) Soil moisture is the essential factor for plants^13,14^, and depends on the total amount of precipitation and its distribution throughout the year, soil water-holding capacity, and evapotranspiration^6^. Trees, for example, reach higher cover with higher rainfall^15,16^; similarly, grasses have greater biomass in areas with more rainfall^10^. (ii) Soil nutrients are primarily determined by the character of bedrock; nutrient rich soils occur typically on basic bedrocks, such as basalt, while nutrient-poor soils prevail on acidic bedrocks, such as granite or sandstone^17^. The prime importance of soil nutrients and their interaction with soil moisture led to the formulation of the PAM-PAN (plant-available moisture—plant-available nutrients) concept, which provides a framework to formalize the structural and functional classification of savannas worldwide^6,14^. (iii) Large herbivores in African savanna represent the last remnants of the world’s megafauna^18–20^. Herbivores affect the balance between the dominance of trees and grasses^2,21,22^ and influence the local diversity of grass species^13^. Moreover, large herbivores, together with fire, prevent savanna from transitioning into a forest^12,15,23,24^. The impact of herbivores depends on their total biomass and the abundance of individual feeding guilds—browsers, grazers, and mixed-feeders^22^. (iv) Fire is the second main factor that reduces plant biomass, after herbivory^20,21^, and interacts with the other two previously mentioned factors, soil moisture and nutrients. Fire frequency increases in areas with more rainfall and less herbivory, a combination resulting in greater biomass production^22^.

The maintenance of biodiversity in savanna is driven by large-scale spatiotemporal dynamics of habitats differing in their position on a gradient of water availability and disturbance^25,26^. Plant communities are defined by the response of the species they harbour to varying levels of the key factors, i.e., some species require high moisture levels and can cope with an intense herbivore pressure^27^, while other species are adapted to arid conditions with occasional fires. Some others will persist along seasonal rivers with large stores of underground water but less severe pressure from large herbivores (especially elephants), who depend on permanently available water^28^.

Here we hypothesize that an important factor affecting savanna biodiversity, including patterns of vegetation richness, is the presence of seasonal rivers that differ in resource provisions, and therefore in animal densities, from perennial rivers or other permanent water sources, such as dams or artificial water points^29^. Seasonal rivers may support relatively lower densities of elephants and other large herbivores, who keep close to water sources and productive environments, especially during dry periods^30,31^. In contrast, perennial rivers experience intense, all-year browsing and trampling pressure by herbivores that may prevent disturbance-sensitive plant species from occurring in such sites. Finally, on crests further away from water sources, disturbances by elephants and other herbivores may be low^29^, but many plant species could be suppressed by drought. Therefore, we predict that plant species richness and diversity, namely that of woody vegetation which forms a large part of elephants’ diet^32^ and is exposed to browsing by other large herbivores, will be greater around seasonal rivers compared to other habitats.

**Figure 1:**
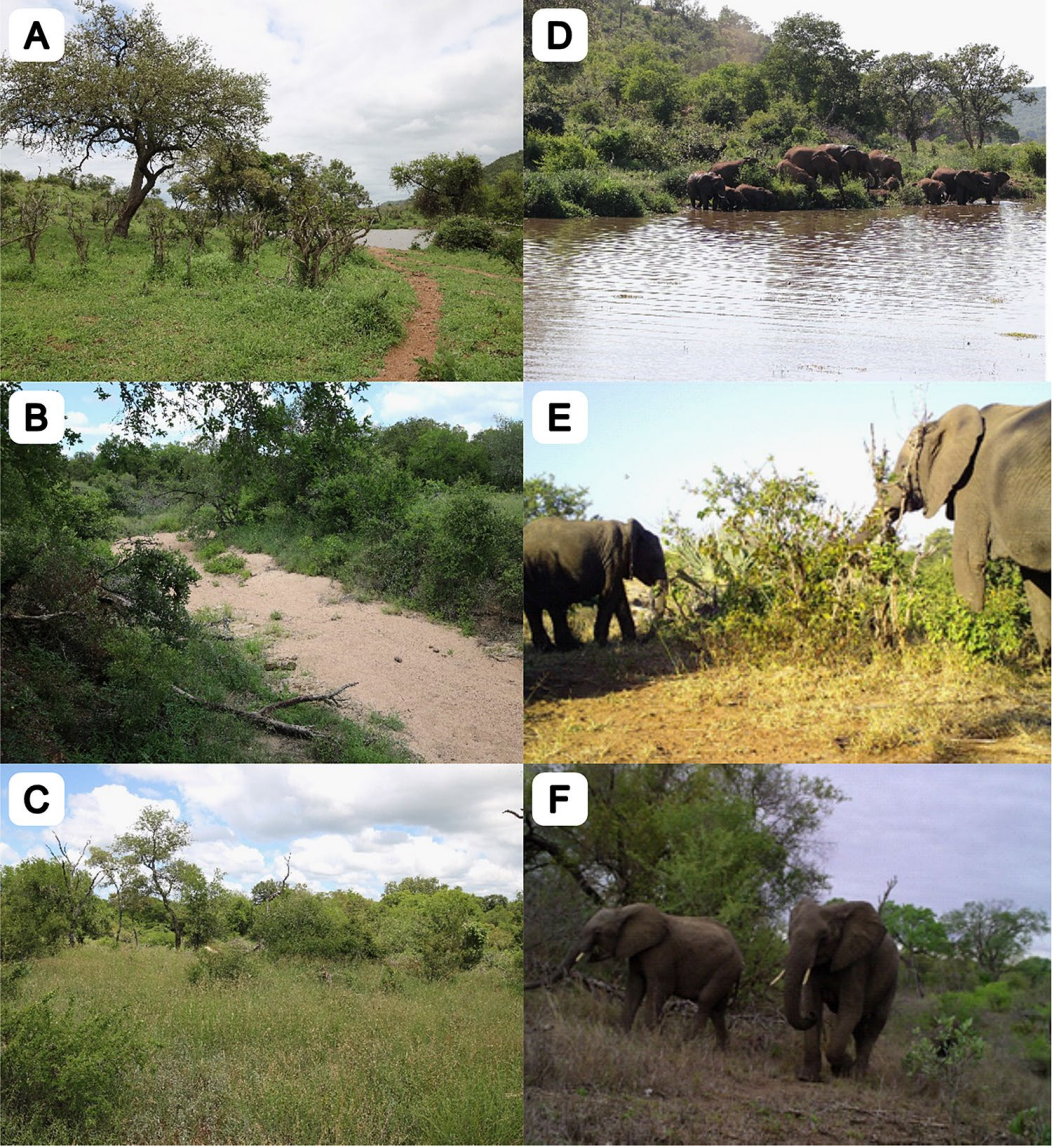
Habitats in the Kruger National Park that were sampled in this study, left column: (**A**) perennial river, (**B**) seasonal river, and (**C**) a dry crest. Right column: Elephants at a perennial plot in rainy (**D**) and dry season (**E**), and at a seasonal river (**F**). Photo: Petr Pyšek (**A**–**C**), Klára Pyšková (**D**–**F**).

**Figure 2:**
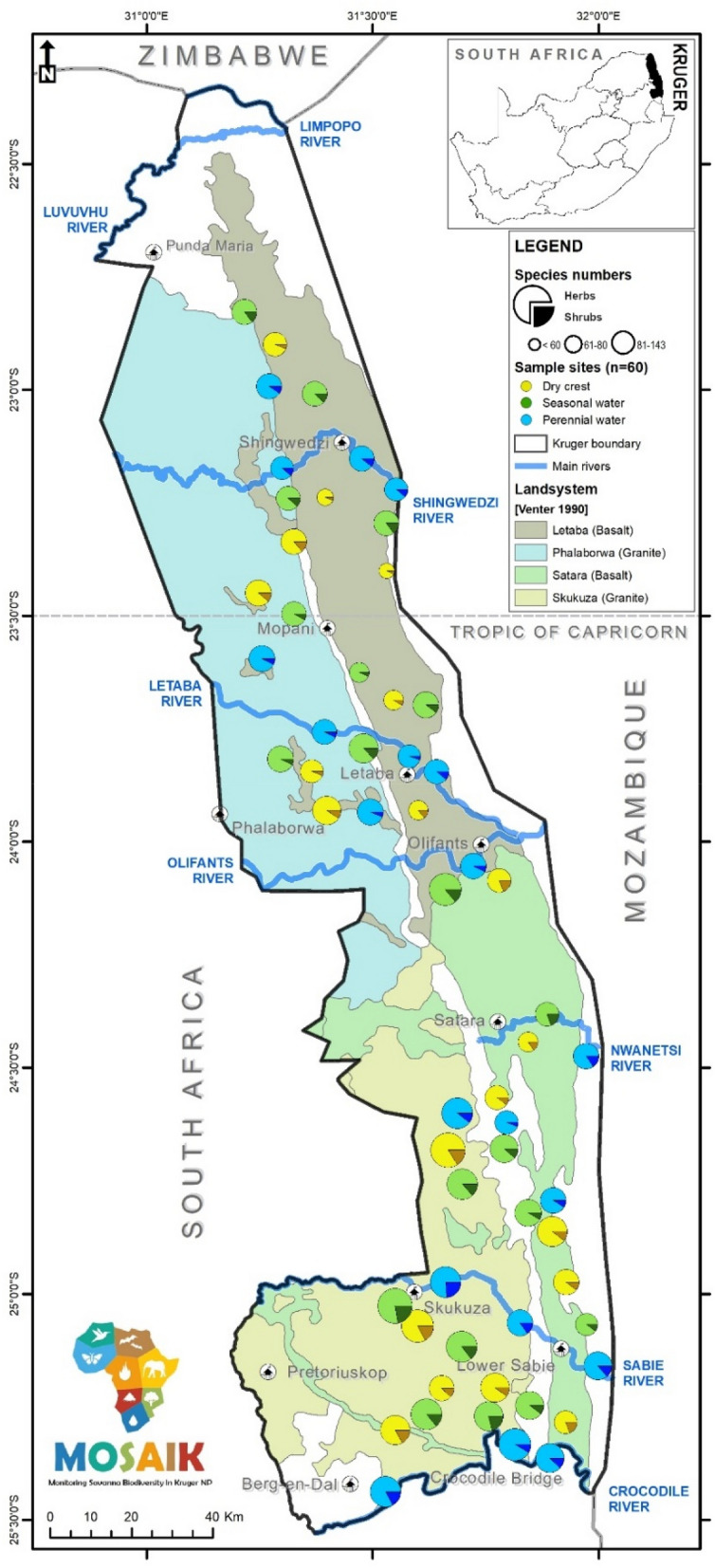
Kruger National Park map with the location of study plots across habitats (dry crest, seasonal river, perennial river) and bedrocks (basalt and granite), reflecting the four most represented landsystems. The total plant species richness in a plot (per 2500 m^2^) and the proportion accounted for by herbs (including grasses) and shrubs is indicated by the size of the circle and shade of the colour, respectively. The figure was created using ArcGIS Desktop, Release 10.4 (Redlands 2011, https://www.esri.com).

To provide insights into factors that drive plant diversity in African savanna and explore their relative importance, we sampled species composition in plant communities, using the Kruger National Park (KNP), South Africa, as a model system. Our study aimed to capture the variation induced by contrasting bedrock types, position on the gradient of water availability, and herbivore presence. To achieve this, we made complete plant inventories in 60 plots of 50 × 50 m, located in three distinct habitats: (i) at perennial rivers, (ii) at seasonal rivers with water available only in the rainy season, and (iii) on crests, at least ~ 5 km away from any water source (Fig. 1), and accounting for the two main substrates in KNP, granites in the western and basalts in the eastern part (Fig. 2). Besides analysing the overall species richness in study plots, we also assessed the extent to which the vegetation in plots represents typical natural vegetation; species that are labelled as ‘important taxa’ for the respective vegetation units^9^ were considered indicator species.

To capture the complexity of determinants of species diversity in KNP, we complemented the analysis of the above-mentioned local factors by exploring the role of large-scale factors. Besides bedrock and distance to waterbodies (as a proxy for water availability), plant richness and diversity are likely to be influenced by factors that can only be detected on large spatial or temporal scales, such as fire history, climate, vegetation characteristics, landscape or anthropogenic disturbance. In addition, a unique feature that needs to be considered in analysing plant species richness in KNP is the pattern of woody species dominance. *Colophospermum mopane,* a shrub or a small tree, is a strong dominant of vegetation in the northern half of KNP; a lower plant diversity generally features this part of the park. The low diversity of other species in the shrub layer can be explained by the direct competition with this dominant species, but mopane was also reported be suppressive to herbs. Timberlake^33^ suggested that the grass layer is consistently poor across the whole range of mopane woodland, generally consisting of *Aristida* and *Eragrostis* species, and the sites exhibit a low alpha diversity, in particular of perennial species. The distribution pattern of mopane in KNP makes its cover and the north–south gradient two heavily confounded factors that need to be taken into account in statistical analyses.

The study was carried out as part of the MOSAIK project (Monitoring Savanna Biodiversity in the Kruger National Park^34^) and aimed at answering the following questions: (i) How does the species richness, diversity, cover, and composition of plant communities respond to differences in water availability and type of bedrock? (ii) Does the effect of water availability and bedrock type differ between herbs and shrubs? (iii) What is the conservation value of plant communities in terms of representativeness of the typical natural vegetation of the study area? (iv) Do the disturbance by herbivores and position on the water availability gradient interact in driving the richness of shrubs? (v) What factors, besides bedrock and water availability, affect the plant richness and cover in KNP? (vi) Does the dominance of *Colophospermum mopane* influence the patterns of plant richness?

## Results

### Overall species richness across plots

In total, we recorded 540 vascular plant species from 67 families at all 60 sampled plots, with Poaceae, Fabaceae, and Malvaceae most represented (99, 53, and 44 species, respectively). Regarding life forms, 381 species were herbs (including 99 grasses), and 139 shrubs or trees (for 20 species, the life form in KNP was ambiguous and not explicitly defined). Plots in perennial- and seasonal-river habitats harboured similar numbers of herb species (286 and 287, respectively), followed by crests (266). Shrub diversity was highest in seasonal rivers with 113 species, while perennial rivers and crests hosted fewer species (106 and 98, respectively). There were 330 species of herbs and 120 shrubs recorded on granite bedrock, with corresponding figures of 316 and 114, respectively, for basalts. In terms of frequency, the most common species were grasses *Panicum maximum* (recorded in 54, i.e., 90% of all plots), *Brachiaria deflexa* (52), *Tragus berteronianus* (49), and *Urochloa mosambicensis* (43), herbs *Phyllanthus maderaspatensis* (47), *Hibiscus micranthus* (41), and *Acalypha indica* (40), and shrubs *Flueggea virosa* (46), *Dichrostachys cinerea* (44), and *Philenoptera violacea* (39). In total, 29 taxa occurred in more than half of the plots sampled (Table 1).

**Table 1:** Plant species that were most frequent in the 60 plots sampled across the Kruger National Park, according to bedrock (granite, basalt)﻿; n = 30 each and habitat type (perennial river, seasonal river, crest); n = 20 each﻿.

Taxon	Family	LH	Total	Granite	Basalt	Perennial	Seasonal	Crest
*Panicum maximum*	Poaceae	pg	54	26	28	17	19	18
*Brachiaria deflexa*	Poaceae	ag	52	26	26	17	18	17
*Tragus berteronianus*	Poaceae	ag	49	24	25	16	17	16
*Phyllanthus maderaspatensis*	Phyllanthaceae	ph	47	27	20	15	15	17
*Flueggea virosa*	Phyllanthaceae	s, t	46	25	21	18	17	11
*Dichrostachys cinerea*	Fabaceae	s, t	44	25	19	14	17	13
*Urochloa mosambicensis*	Poaceae	pg	43	20	23	13	15	15
*Hibiscus micranthus*	Malvaceae	s	41	24	17	13	16	12
*Aristida adscensionis*	Poaceae	ag	40	19	21	14	13	13
*Acalypha indica*	Euphorbiaceae	ah	40	21	19	15	18	7
*Philenoptera violacea*	Fabaceae	t	39	21	18	15	16	8
*Talinum caffrum*	Talinaceae	ph	38	24	14	11	14	13
*Phyllanthus reticulatus*	Phyllanthaceae	s, t	38	19	19	13	12	13
*Tephrosia purpurea*	Fabaceae	ph	37	18	19	14	11	12
*Abutilon angulatum* var. *angulatum*	Malvaceae	ph, s	37	22	15	15	15	7
*Eragrostis superba*	Poaceae	pg	36	20	16	13	11	12
*Grewia bicolor*	Malvaceae	s, t	36	21	15	16	11	9
*Digitaria eriantha*	Poaceae	pg	35	20	15	4	15	16
*Heliotropium nelsonii* var. *steudneri*	Boraginaceae	ph	34	18	16	12	13	9
*Ceratotheca triloba*	Pedaliaceae	ah	33	17	16	9	13	11
*Tribulus terrestris*	Zygophyllaceae	ah	33	19	14	11	13	9
*Cissus cornifolia*	Vitaceae	s	32	18	14	10	6	16
*Enneapogon cenchroides*	Poaceae	ag	32	15	17	13	12	7
*Cenchrus ciliaris*	Poaceae	pg	31	14	17	8	14	9
*Combretum hereroense*	Combretaceae	t	31	20	11	9	14	8
*Corbichonia decumbens*	Lophiocarpaceae	ah, ph	31	21	10	12	12	7
*Maerua parvifolia*	Capparaceae	s	31	20	11	13	12	6
*Hibiscus sidiformis*	Malvaceae	ah	31	18	13	13	13	5
*Combretum mossambicense*	Combretaceae	s,t	31	13	18	13	14	4

Numbers of records for taxa that occurred in more than half of all the plots are shown. Life history (LH): ag = annual tufted grass, pg = perennial tufted grass, ah = annual herb, ph = perennial herb, s = shrub, t = tree.

### Main effect of bedrock

**Figure 3:**
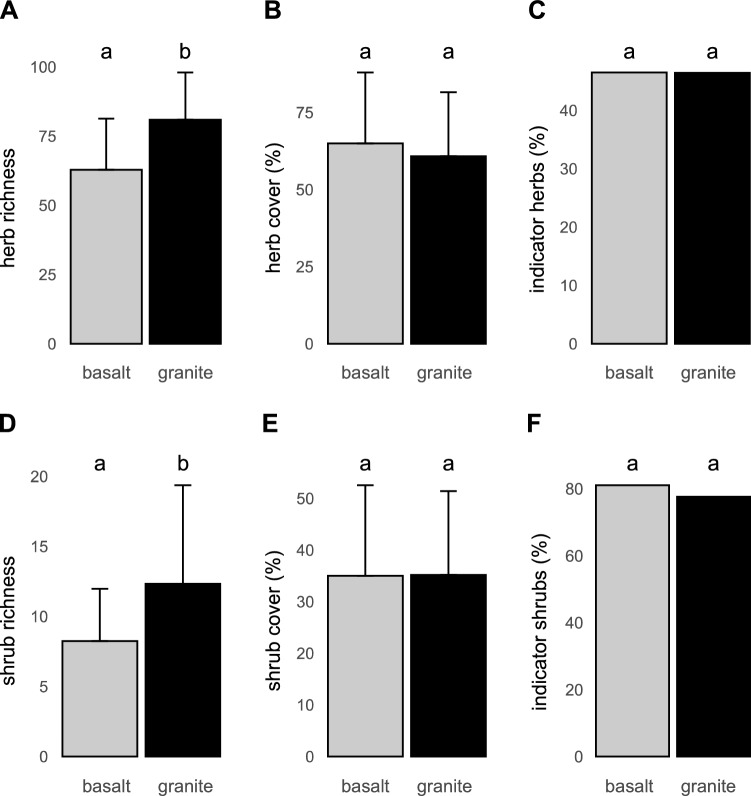
Richness, cover and proportion of indicator species of herbs and shrubs on granitic and basaltic bedrock. Bars show means for each type of bedrock (n = 30) and error bars are associated with standard deviation (SD). The bold letters above bars indicate significant differences between bedrocks (including marginally significant, 0.05 < *p* < 0.1, in normal font).

The results of statistical analyses are summarized in Table 2. Plots on granite harboured more herbs and shrubs than those on basalt (Fig. 3A, D; mean ± SD: 80.9 ± 16.8 and 12.3 ± 6.9 vs. 62.8 ± 18.2 and 8.2 ± 3.7; *p* = 0.001 and *p* = 0.0756, respectively). Similarly, plots on granite had a higher Shannon diversity H’ of herbs than basalt plots (2.12 ± 0.60 vs. 1.65 ± 0.65; *p* = 0.0113, respectively; note that due to similar results of analyses of species richness and Shannon H’ only the former are presented graphically). Plots on granite had more indicator species of herbs and shrubs compared to plots on basalt (37.2 ± 7.9 and 8.8 ± 3.8 vs. 28.5 ± 8.4 and 6.4 ± 2.7; *p* = 0.001 and *p* = 0.0697, respectively). However, neither herb and shrub cover (Fig. 3B, E), nor the proportions of indicator species significantly differed between bedrocks (Fig. 3C, F).

**Table 2:** Summary table of the differences in species richness, diversity, cover, and representation of indicator species recorded in the study plots by habitat and bedrock, with results of statistical tests.

Characteristic	Life form	Bedrock	Habitat	Bedrock × Habitat
Species richness (no. of species per plot)	Herbs	*p* = 0.001, T = 3. 917	*p* = 0.001, T = 3.532	*p* = 0.018, T = − 2.485
Species richness (no. of species per plot)	Shrubs	*p* = 0.075, T = 1.885	*p* = 0.081, T = 1.798	*p* = 0.408, T = − 0.837
Species diversity (Shannon index H’)	Herbs	*p* = 0.011, T = 2.822	*p* = 0.003, T = 3.132	*p* = 0.244, T = − 1.185
Species diversity (Shannon index H’)	Shrubs	*p* = 0.483, T = 0.716	*p* = 0.066, T = 1.894	*p* = 0.572, T = 0.570
Community cover (%)	Herbs	*p* = 0.105, T = − 1.71	*p* = 0.014, T = − 2.58	*p* = 0.087, T = 1.76
Community cover (%)	Shrubs	*p* = 0.339, T = 0.98	*p* = 0.08, T = 1.8	*p* = 0.014, T = − 2.60
Indicator species (% of the total)	Herbs	*p* = 0.982, T = − 0.023	*p* = 0.032, T = − 2.225	*p* = 0.793, T = − 0.265
Indicator species (% of the total)	Shrubs	*p* = 0.401, T = − 0.861	*p* = 0.035, T = − 2.185	*p* = 0.487, T = 0.703
Relative cover	Grass	*p* = 0.072, T = − 1.911	*p* = 0.0003, T = − 4.022	*p* = 0.377, T = 0.895

DF_res_ = 18 for bedrock, DF_res_ = 36 for Habitat, and Bedrock × Habitat interaction.

### Main effect of habitat

**Figure 4:**
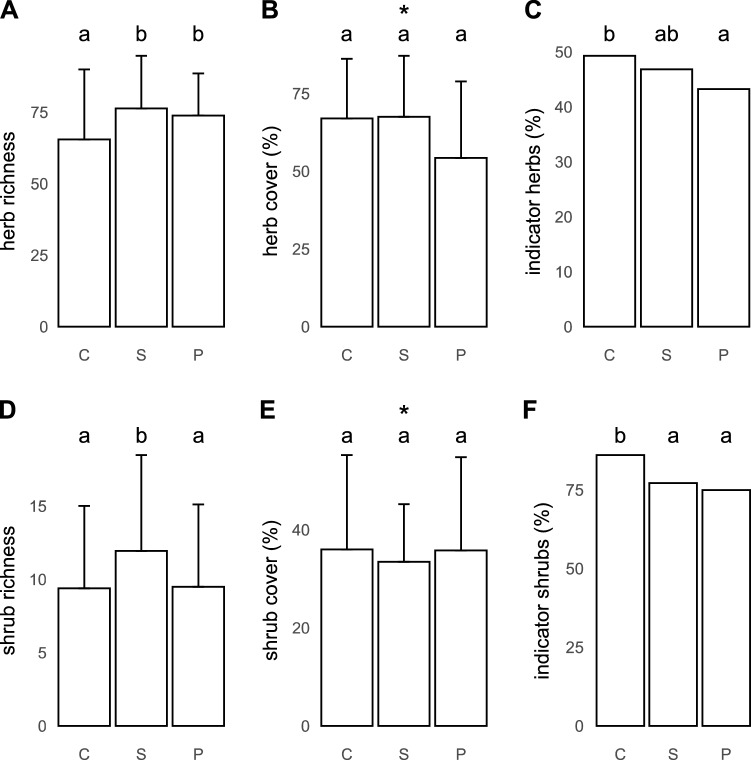
Plant species richness, cover, and share of indicator species of herbs and shrubs on three plot types—crests (C), seasonal rivers (P), and perennial rivers (P). Bars show means for each type of bedrock (n = 20), and error bars show the associated standard deviation (SD). The letters above bars indicate significant differences among the three types of plots (including marginally significant, 0.05 < *p* < 0.1). Asterisks indicate the significance of the overall model when pairwise contrasts were not significant.

Crests were poorer in herb species than both seasonal and perennial rivers (Fig. 4A; 65.5 ± 23.9, 76.4 ± 17.9 and 73.9 ± 14.4, respectively; *p* = 0.021 and *p* = 0.09), and seasonal rivers harboured marginally significantly more shrubs (Fig. 4D; 12.0 ± 6.4, *p* = 0.095 and *p* = 0.056, respectively) than perennial rivers (9.5 ± 5.5), and crests (9.4 ± 5.5). Plots near perennial rivers showed the highest Shannon diversity H’ of herbs and shrubs (2.29 ± 0.50 and 0.67 ± 0.47; *p* = 0.0034 and *p* = 0.0663, respectively, for the overall models).

Herbs reached a higher cover at seasonal rivers and on crests than at perennial rivers (Fig. 4B; 67.5%, range 30–95%; 67.0%, range 30–100%; and 54.3%, range 20–100%, respectively, *p* = 0.0141 for the overall model). The cover of shrubs on crests and at perennial rivers was 36%, (Fig. 4E; range 10–75% and 0.5–75%, respectively), significantly higher than at seasonal rivers, albeit marginally (33.5%, range 20–60%, *p* = 0.08). Crests harboured the greatest proportions of indicator species of herbs and shrubs (Fig. 4C,F; herbs: 49.3%, range 34.0–62.2%, and shrubs: 86%, range 52.3–100%; *p* = 0.0324 and *p* = 0.0354, respectively, for the overall models).

### Interaction between bedrock and habitat

**Figure 5:**
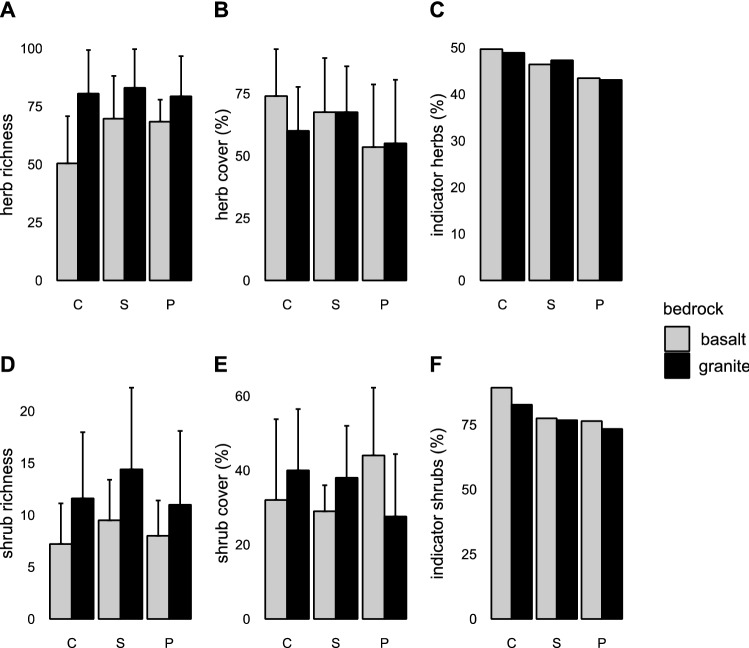
Plant species richness, cover, and share of indicator species of herbs and shrubs on three plot types—crests (C), seasonal rivers (S), and perennial rivers (P) and on two types of bedrocks (granite and basalt). Bars show means for each type of bedrock and habitat combination (n = 10), and error bars show associated standard deviation (SD).

Crests on granite harboured more herbs than crests on basalt (Fig. 5A; 80.5 ± 17.8 vs. 50.4 ± 19.3, respectively; *p* = 0.0178).

On granites, herbs reached the highest cover along seasonal rivers, but on basalts, their cover was highest on crests, the difference being marginally significant (Fig. 5B; 67.5%, range 40–95%, vs. 74.0%, range 40–100%, respectively; *p* = 0.0872). On granites, shrubs had a higher cover on crests and at seasonal rivers than at perennial rivers (Fig. 5E; 40.0%, range 15–60%; 38.0%, range 20–60%; and 27.6%, range 0.5–60%, respectively), but on basalts, shrubs reached the highest cover at perennial rivers (44.0%, range 20–75%; *p* = 0.0135).

On granites, vegetation on crests and at seasonal rivers contained more indicator species of herbs compared to perennial rivers (Fig. 5C; 38.8 ± 7.9 and 38.5 ± 5.2 vs. 34.2 ± 9.2, respectively), but on basalts, plots at perennial and seasonal rivers harboured more herb indicator species than plots on crests (29.1 ± 4.5 and 32.4 ± 9.8 vs. 24.1 ± 7.9, respectively; *p* = 0.0512 for the overall models on the effects of bedrock, habitat type and their interactions). The proportions of indicator shrub species in different habitats did not significantly differ between the two bedrocks (Fig. 5F).

### Effects of bedrock and habitat on grass versus herb covers

**Figure 6:**
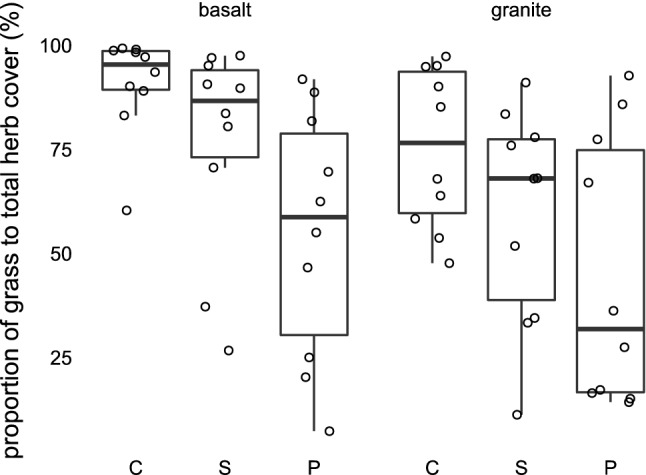
The proportion of grass cover shown for particular bedrocks (granite, basalt) and habitats (C—crest, S—seasonal river, P—perennial river). Based on the sums of percentage covers of all species in the herb layer of a plot, expressed separately for grasses and herbs.

The relative cover of grasses differed among bedrocks and habitats (*p* = 0.0721, and *p* = 0.0003, respectively), with higher values recorded on basalt (74%, range 7–99%) than granite (60%, range 11–97%) (Fig. 6). Concerning the type of habitat, the largest relative cover of grasses was recorded on crests (83%, range 48–99%), differing significantly from perennial rivers (50%, range 7–93%, *p* = 0.0001) and marginally significantly from seasonal rivers (68%, range 11–98%, *p* = 0.055). Perennial rivers also marginally significantly differed from seasonal rivers in their relative grass cover (*p* = 0.056).

### Effects of bedrock and habitat on species composition

The most common species on particular bedrocks and in the habitats sampled are listed in Table 1. The multivariate ordination of data showed that the composition of herb communities differed between granites and basalts, but the difference was only marginally significant (*p* = 0.094); the species composition of shrubs did not differ between the two bedrocks (*p* = 0.13).

**Figure 7:**
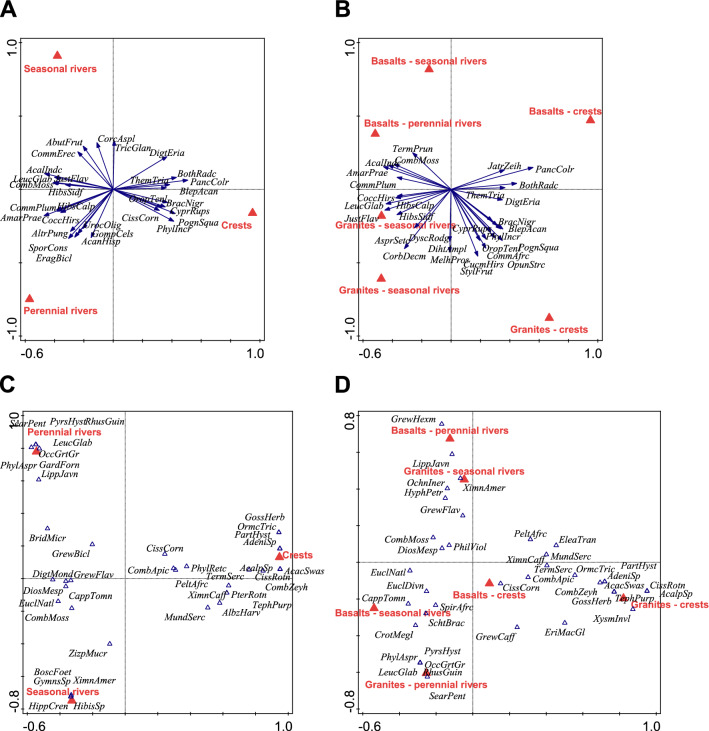
Ordination diagrams of the vegetation data showing the effect of habitat on species composition (**A**, **C**) and that of habitat × bedrock interaction (**B**, **D**) separately for herbs (including grasses; top panels **A**, **B**) and shrubs (bottom panels **C**, **D**). The models with herb species as responses (**A**, **B**) were created using the linear method (RDA), while the ordinations with shrubs as responses (**C**, **D**) were created using the unimodal models. Species codes: AbutFrut—*Abutilon fruticosum*, AcacSwas—*Acacia swasica*, AcalIndc—*Acalypha indica*, AcalpSp—*Acalypha* sp., AcanHisp—*Acanthospermum hispidum*, AdeniSp—*Adenium* sp., AlbzHarv—*Albizia harveyi*, AltrPung—*Alternanthera pungens*, AmarPrae—*Amaranthus praetermissus*, AsprSetc—*Asparagus setaceus*, BlepAcan—*Blepharis acanthoides*, BoscFoet—*Boscia foetida*, BothRadc—*Bothriochloa radicans*, BracNigr—*Brachiaria nigropedata*, BridMicr—*Bridelia micrantha*, CappTomn—*Capparis tomentosa*, CissCorn—*Cissus cornifolia*, CissRotn—*C. rotundifolia*, CoccHirs—*Cocculus hirsutus*, CombApic—*Combretum apiculatum*, CombMoss—*C. mossambicense*, CombZeyh—*C. zeyheri*, CommAfrc—*Commiphora africana*, CommErec—*Commelina erecta*, CommPlum—*Commicarpus plumbagineus*, CorbDecm—*Corbichonia decumbens*, CorcAspl—*Corchorus aspleniifolius*, CrotMeg—*Croton megalobotry*s, CucmHirs—*Cucumis hirsutus*, CyprRups—*Cyperus rupestris*, DigtEria—*Digitaria eriantha*, DigtMond—*D. monodactyla*, DihtAmpl—*Diheteropogon amplectens*, DiosMesp—*Diospyros mespillifera*, DyscRodg—*Dyschoriste rodgersii*, EleaTran—*Eleaeodendron transvaalense*, EragBicl—*Eragrostis bicolor*, EriMacGl—*Eriospermum mackenii* subsp. *galpinii*, EuclNatl—*Euclaea natalensis*, GardForn—*Gardenia foranense*, GompCels—*Gomphrena celosioides*, GossHerb—*Gossipium herbaceum*, GrewBicl—*Grewia bicolor*, GrewCaff—*G. caffra*, GrewFlav—*G. flavescens*, GrewHexm—*G. hexamita*, GymnSp—*Gymnosporia* sp., HibisSp—*Hibiscus* sp., HibsCalp—*H. calyphyllus*, HibsSidf—*H. sidiformis*, HippCren—*Hippocratea crenata*, HyphPetr—*Hyphaene petersiana*, JatrZeih—*Jatropha zeiheri*, JustFlav—*Justicia flava*, LeucGlab—*Leucas glabrata*, LippJavn—*Lippia javanica*, MelhPros—*Melhania prostrata*, MundSerc—*Mundulea sericea*, OccGrtGr—*Occimum gratissimum* var. *gratissimum*, OchnIner—*Ochna inermis*, OpunStrc—*Opuntia stricta*, OrmcTric—*Ormocarpum trichocarpum*, OropTenl—*Oropetium tenellus*, PancColr—*Panicum coloratum*, PartHyst—*Parthenium hysterophorus*, PeltAfrc—*Peltophorum africanum*, PhilViol—*Philenoptera violacea*, PhylAspr—*Phyllanthus asperulatus*, PhylIncr—*P. incurvus*, PhylRetc—*P. reticulatus*, PognSqua—*Pogonarthria squarrosa*, PterRotn—*Pterocarpus rotundifolius*, PyrsHyst—*Pyrostria hystrix*, RhusGuin—*Rhus guinense*, SearPent—*Searsia penthriri*, SporCons—*Sporolobus consimilis*, StylFrut—*Stylosanthes fruticosa*, TephPurp—*Tephrosia purpurea*, TermPrun—*Terminalia prunoides*, TermSerc—*T. sericea*, ThemTria—*Themeda triandra*, TricGlan—*Tricliceras glanduliferum*, UrocOlig—*Urochloa oligotricha*, XimnAmer—*Ximenia americana*, XimnCaff—*X. caffra*, XysmInvl—*Xysmalobium involucratum*, ZizpMucr—*Ziziphus mucronata*.

In contrast, the composition of both herbs and shrubs significantly differed among habitats (*p* = 0.002 and *p* = 0.002, respectively). For herbs, the crests differed from both seasonal and perennial rivers, and perennial rivers differed from seasonal rivers. For shrubs, crests significantly differed from seasonal and perennial rivers, but seasonal rivers did not differ significantly from perennial rivers. In the ordination plot, the first axis was associated with the presence of a river (as opposed to dry crest), the second with its type, separating seasonal and perennial rivers. Herb species such as *Acanthospermum hispidum*, *Alternanthera pungens,* and *Amaranthus praetermissus* were typically associated with perennial river plots, *Abutilon fruticosum*, *Corchorus asplenifolius,* and *Panicum maximum* with seasonal rivers, and *Brachiaria nigropedata*, *Phyllanthus incurvus,* and *Pogonarthria squarrosa* were typically found on crests (Fig. 7A). Shrubs *Lippia javanica*, *Searsia gueinzii,* and *S. pentheri* were typically found at perennial rivers, *Hippocratea crenata* and *Ximenia americana* at seasonal rivers, and *Combretum zeyheri*, *Ormocarpum trichocarpum,* and *Terminalia sericea* on crests (Fig. 7B).

Of the total 540 species recorded, 48.0% occurred in all three habitats, and 25.2% were specific to one of them. From the life-history perspective, similar proportions of forbs, grasses and woody species shared all three habitats (46.1%, 51.5% and 51.1%, respectively). Still, seasonal rivers harboured the highest number of woody species that occurred exclusively in that habitat (14, i.e. 10.1% of the total), as compared to perennial rivers (12 species, i.e. 8.6%) and crest (6 species, i.e. 4.3% of all woody species). In contrast, perennial rivers were the richest in forbs recorded exclusively in that habitat (31, corresponding to 11.0% of all forbs) (Supplementary Fig. 1).

For both herbs and shrubs, a significant interaction between bedrock and habitat was detected (*p* = 0.002 and *p* = 0.008, respectively), indicating that species associated with different habitats reflecting water availability also differed between the two bedrocks. For example, *Jatropha zeyheri, Panicum coloratum,* and *Zanthoxylum humile* are associated with crests on basalt, while *Commiphora africana*, *Cucumis hirsutus* and *Pogonarthria squarrosa* with crests on granite (Fig. 7C). Similarly, the shrubs *Cissus cornifolia*, *Euclea divinorum,* and *Spirostachys africana* preferred basaltic crests while *Gossypium herbaceum*, *Ormocarpum trichocarpum,* and *Vachellia swazica* crests on granite (Fig. 7D).

### Herbivore abundances by habitats

The number of herbivores (measured as the number of all species’ records by 60 camera traps) over 140 days differed significantly between habitats (p < 0.001), with highest numbers per plot recorded at perennial rivers (2266.4 ± 1634.7), lowest at the crest (293.6 ± 230.0), and intermediate at seasonal rivers (1077.4 ± 820.6).

### Effect of large-scale factors

**Figure 8:**
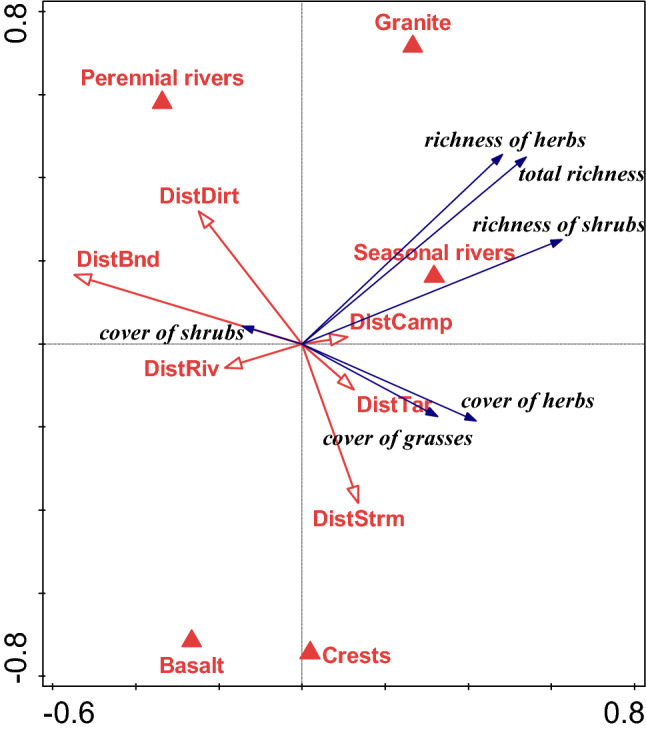
The relationships of the large-scale factors and measures of plant richness and cover in the sampled plots after filtering out the effects of (i) bedrock (basalt, granite), water availability (perennial river, seasonal river, crest) and arrangement of individual plots into triplets; and (ii) the spatial arrangement of plots (expressed by the PCO1, PCO2 and PCO3 coordinates). The correlations between individual variables are expressed by the directions of the corresponding arrows. Arrows aiming in the same or similar directions are positively related, while variables represented by arrows aiming in the opposite directions are negatively related. Rectangular angle between two arrows shows no relation, at least in the dimensionality shown by the plot.

The ordination model (RDA) for plant species richness and cover with all large-scale predictors (see Supplementary Table 1), and bedrock and habitat type as covariables yielded non-significant results on canonical axes, either with or without covariables representing the spatial effects (Table 3). However, the forward selection identified mean temperature, variation in Enhanced Vegetation Index, and the distance from dirt roads as the most important predictors. Accordingly, the direct gradient ordination model (RDA) with these predictors was highly significant (*p* = 0.002) and remained so with the spatial effects accounted for (*p* = 0.008; Fig. 8).

**Table 3:** Results of the ordination models (direct gradient analysis—RDA) exploring and testing the relations between large-scale predictors and plant species richness/cover in the Kruger National Park.

Forward selection	p-value (without spatial effects )	Explained variance (%)	p-value (with spatial effects)	Explained variance (%)
**Mean temperature (tempMean)**	**0.006**	20.1	-	-
**Variation in Enhanced Vegetation Index (eviSD)**	**0.032**	5.8	-	-
**Distance from gravel roads (distDirt)**	**0.002**	7.3	-	-
Covariables: bedrock, habitat			**-**	**-**
**tempMean, eviSD, distDirt**	**0.002**	30.5	**0.008**	12.7
All predictors	0.118	26.7	0.182	10.9
Rain^1^	0.158	8.4	0.588	-0.3
*Distances*^2^	0.472	3.8	*0.086*	7.4
**Temperature**^3^	**0.008**	27	**0.006**	10.2
Surface water^4^	0.846	− 2.6	0.304	0.2
Enhanced Vegetation Index (EVI)^5^	0.078	12.1	0.162	2.8
Fire^6^	0.998	− 3.5	0.68	− 0.8

In all ordination models, total plant richness, richness of herbs, richness of shrubs, cover of grasses and cover of shrubs were used as the response variables. Spatial effects are represented by the three main principle coordinates (PCO1, PCO2, PCO3). Significant effects are displayed in bold, marginally significant effects in italics. The significance values refer to individual predictors in case of forward selection, and to the significance of all canonical axes in the remaining ordination models. See Supplementary Table 1 for detailed description of variables.

^1^Summary effect of rainfall (rainSum, rainMean, rainSD).

^2^Summary effect of distance from potential sources of propagules—roads, rivers, rest camps and KNP boundary (DistBnd, distCamp, distTar, distDir, distRiv, distStrm; see Supplementary Table 1).

^3^Summary effect of temperature and its variation over time (tempMean, tempSD, tempMin, tempMax).

^4^Summary effect of surface water occurrence (waterSum, waterMean, waterSD).

^5^Summary effect of Enhanced Vegetation Index (eviSum, eviMean, eviSD).

^6^Summary effect of fire (FireSum, FireMean, FireSD).

The effect of temperature-related variables on plant species richness and covers was highly significant (*p* = 0.008) and remained so with the spatial effects included (*p* = 0.006). The effect of distances was non-significant but turned marginally significant after including the spatial effects (*p* = 0.086; Table 3).

In the linear regression models with residuals from the linear mixed-effect models (LMM) as responses, the total plant richness and herb richness were negatively and significantly related to all four measures used to characterize the long-term temperature, except for the total plant richness being marginally significantly related to temperature minima and maxima (*p* = 0.057 and 0.062, respectively). All significant relationships turned to be marginally significant after including the spatial effects into the LMM model (Table 4). Besides temperature, there were marginally significant effects of other variables on grass and herb covers in the LMM models; grass cover decreased with the distance of the plot from a gravel road (*p* = 0.092 and *p* = 0.08 with and without spatial effects, respectively). With spatial effects included, herb cover was positively and marginally significantly related to the variation in the Enhanced Vegetation Index (*p* = 0.082; Table 4). It should be also noted that the amount of variance in the data explained by the large-scale predictors is generally low, even in case of predictors with significant effects (Table 4), suggesting that the effects of large-scale predictors are limited in comparison with the strong local-scale factors.

**Table 4:** The results of univariate regression models testing the relationships between the large-scale predictors, identified as significant by the ordination models (Table 3), and measures of plant species richness and cover in plots in the Kruger National Park.

Response	Predictor	*p* value (without spatial effects)	Estimate (without spatial effects)	Explained variability (adjusted R-square)	p-value (with spatial effects)	Estimate (with spatial effects)	Explained variability (Adjusted R-square)
Richness (total)	tempMean	**0.043**	− 1.6736	0.053	*0.083*	− 1.474	0.035
Richness (total)	tempMin	*0.0567*	− 3.439	0.045	*0.078*	− 3.257	0.036
Richness (total)	tempMax	*0.0620*	− 0.8389	0.043	*0.086*	− 38.43	0.034
Richness (total)	tempSD	**0.0496**	− 26.07	0.049	*0.088*	− 23.27	0.033
Richness (shrubs)	tempMean	0.161	− 0.03676	0.017	0.271	− 0.030	0.004
Richness (shrubs)	tempMin	0.129	− 0.08666	0.023	0.196	− 0.076	0.012
Richness (shrubs)	tempMax	0.548	− 0.008596	− 0.011	0.686	− 0.2903	− 0.014
Richness (shrubs)	tempSD	0.290	− 0.4469	0.002	0.410	− 0.3595	− 0.005
Richness (herbs)	tempMean	**0.0243**	− 1.7712	0.069	*0.076*	− 1.436	0.037
Richness (herbs)	tempMin	**0.0352**	− 3.615	0.058	*0.0625*	− 3.272	0.042
Richness (herbs)	tempMax	**0.0297**	− 0.9278	0.063	*0.05*	− 41.50	0.048
Richness (herbs)	tempSD	**0.0241**	− 28.45	0.069	*0.0636*	− 24.01	0.064
Cover (grasses)	eviSD	0.389	7.103	− 0.004	0.288	16.08	0.003
Cover (grasses)	distDirt	*0.0804*	− 0.6234	0.035	*0.092*	− 0.258	0.032
Cover (herbs)	eviSD	0.173	1.30661	0.015	*0.082*	1.740	0.035
Cover (herbs)	distDirt	0.342	− 0.02303	− 0.001	0.609	− 0.005	− 0.013
Cover (herbs)	eviSD	0.493	− 0.58314	− 0.009	0.493	− 0.583	− 0.009
Cover (herbs)	distDirt	0.534	− 0.01333	− 0.01	0.597	− 0.005	− 0.012

For each response variable, values from models not considering spatial effects as well as those with significant principal coordinates (PCO1, PCO2, PCO3) included to account for spatial effects are presented. Significant effects are displayed in bold, marginally significant in italics. See Supplementary Table 1 for description of predictor variables.

### Effect of *Colophospermum mopane* cover

The marginal effects, i.e. not considering the confounding factors, of the mopane cover and north–south gradient on total, herb, and shrub species richness were significant (Table 5). The partial effects of the mopane cover (after excluding the effect of the north–south gradient) on all three measures of plant richness were not significant, but the partial effect of the north–south gradient (after excluding the effect of mopane cover) was significant, with *p* = 0.001, *p* = 0.004, and *p* = 0.011 for the total, herb and shrub species richness, respectively. After excluding the effects of bedrock, habitat, and triplet as a grouping variable, the partial effects of mopane cover were not significant for total species richness, but marginally significant for herbs and shrubs (*p* = 0.095 and *p* = 0.059, respectively). The partial effects of the north–south gradient (excluding the effects of bedrock, habitat, and triplet as a grouping variable) were marginally significant for total species richness and significant for herbs (*p* = 0.077 and *p* = 0.049, respectively), and non-significant for shrubs.

**Table 5:** The results of variance partitioning between the effects of mopane (*Colophospermum mopane*) cover and the north–south (N–S) gradient in the Kruger National Park on plant species richness.

Response	Predictor	Marginal effect	Estimate	Effect	Explained variability (adjusted R-square)	Partial effect (N-S × mopane)	Estimate	Effect	Explained variability (adjusted R-square)	Partial effect (bedrock × habitat)	Estimate	Effect	Explained variability (adjusted R-square)
Species richness (total)	Mopane cover	**0.0095**	− 26.30	−	0.095	0.895	− 1.13	−	− 0.017	0.123	− 7.217	−	0.024
Species richness (total)	N–S gradient	**< 0.001**	16.77	+	0.313	**0.0013**	111.08	+	0.151	*0.0767*	3.072	+	0.037
Species richness (herbs)	Mopane cover	**0.0219**	− 19.83	−	0.071	0.951	− 0.47	−	− 0.018	*0.0946*	− 7.462	−	0.031
Species richness (herbs)	N–S gradient	**< 0.001**	12.85	+	0.253	**0.0040**	8.73	+	0.119	**0.0494**	3.246	+	0.049
Species richness (shrubs)	Mopane cover	**0.0002**	− 1.36	−	0.195	0.169	− 0.49	−	0.016	*0.0585*	− 0.37167	−	0.044
Species richness (shrubs)	N–S gradient	**< 0.001**	0.61	+	0.288	**0.0106**	0.33	+	0.092	0.115	0.11582	+	0.026

The marginal effects represent the coarse effects, without accounting for the confounded factors, while partial effects represent the net effects after accounting for the confounded factors. Significant effects are in bold, marginally significant in italics, the direction of the effect is indicated by the plus/minus symbol.

## Discussion

### Determinants of plant species richness: interaction of bedrock and habitat

Our study is based on the sampling of the complete floristic composition, both woody plants and herbs, of plant communities in those Kruger National Park habitats that are assumed to underlie the major gradient of variation in plant species richness and composition. The robustness of the data is illustrated by the fact that with the 540 taxa recorded in our plots, we captured over a quarter of the total plant diversity of KNP (1903 taxa in Eckhardt et al.^35^; most recent number: ~ 2000 taxa following G. Zambatis et al., unpublished data), even though the total area of the plots represented less than 1/100,000 of the total area of KNP.

Savannas are inherently heterogeneous ecosystems driven by complex interactions between physical landscape properties, rainfall, herbivores and fire to produce dynamic landscape patterns^5,36,37^. Correspondingly, we found that bedrock and water availability affect plant species richness, diversity, and composition of savanna communities in KNP. We found that the vegetation on granites is richer in species and more diverse than that on basalts, which have a more varied physical landscape template^37^ but more fertile soils^38,39^. We suggest that the low number of species on basalts occur because of less favourable conditions, in terms of water supply and mechanical stress, for most plant species. Different soil types develop on each bedrock—clayey soils are typically formed by the weathering of basalts, while sandy soils are formed on granites. At low rainfall, clayey soils are relatively drier for plants than sandy ones because their permanent wilting point occurs at higher soil water content than at sandy soils^40,41^. Further, the reduced infiltration on clays results in quick runoff during heavy rains and the inability to store water in soil^9^; shallow water penetration is more favourable for grasses, whose root zones are located in the upper soil layers^42^. In addition, basalts weather into more fertile soils than granites^38,39^, which contributes to the dominance of C4 grasses^17^, and in turn, may result in low plant diversity on this bedrock. Low water requirements of C4 grasses enable them to coexist with trees, the strongest competitors for light from the plant kingdom, whose competitive ability is reduced under limited water supply^15,43,44^. Lastly, soils differ in relation to geomorphology especially on the granitic bedrock; these catenas include coarse sandy soils on crests and clayey soils at the footslopes and river bottoms. Soil sequences primarily depend on terrain modulation and character of watercourse network; they are more developed in hilly landscapes with big rivers^17^. In our study, we did not collect soil samples, thus we were unable to classify soils and assess their effect on plants. Most of our plots were located either on flat valley bottoms close to rivers or on flat crests. Therefore, we believe that soil variation in our plots could be roughly described as a combination of habitat type and bedrock (i.e. coarse sandy soils found typically on granite crests and fine clayey soils on basaltic crests). Nevertheless, the character of soil on plots near perennial and seasonal rivers may have been influenced, besides the bedrock, also by the alluvial effects.

Besides, the effect of bedrock interacts with that of water availability; this is most strongly manifest by crests on basalts that host the lowest numbers of herbs, and their species poverty can be attributed to the combined effect of mechanical stress, drought, and competition of dominant grasses^24^. Desiccation of basaltic soils, leading to large cracks, may also damage the roots mechanically^45^. Such mechanical stress may favour the dominance of grasses, which have many fibrous roots, and vertical cracks damage only part of them, while forbs with a single taproot may be damaged more seriously^9^. The strong dominance of grasses on basalts also reduces space and resource capacity for other species, thereby decreasing richness and diversity. In our basalt plots, grasses made up on average 74% of the herb layer cover, markedly more than on granite, where the relative cover of grasses reached 60%. Accordingly, the basaltic crests also hosted the lowest numbers of indicator species typical for given vegetation units (as defined in Mucina and Rutherford^9^).

Species’ cover showed a pattern different from that of species richness and diversity: herbs, including grasses, reached the highest cover on basaltic crests, where their species richness was lowest of all habitats. The high grass cover on basalts may be explained by their fast recovery after heavy grazing due to a high amount of nutrients in soil on this bedrock (in contrast to granites that are poor in nutrients and the recovery is slower^35,46^) and C4 metabolism of grasses that enables them to photosynthesize more effectively under dry and hot conditions than in C3 forbs^47^. This may suggest that the diversity of herbs is limited by the dominance of a few grass species rather than by environmental stress, but most likely, these two factors act in concert—mechanical and physiological stress acts as an environmental filter, selecting few grass species that reach dominance and further reduce the richness and diversity of other species.

### Structure of plant communities: differences in species composition

Only marginally significant differences in the species composition of herbs were detected between the two types of bedrocks. This result is somewhat unexpected, as acidophilous floras usually differ substantially from basiphilous floras worldwide^48^. However, the results from KNP suggest that the two bedrocks share most species; the vegetation on basalts represents an impoverished version of that on granite, without a major qualitative difference—the basaltic community is “nested” within the granitic community (Supplementary Fig. 1).

On the contrary, compositional differences between the three habitats were significant for herbs as well as for shrubs. In general, plots near perennial rivers are rich in species but host many ruderal weeds or naturalized aliens, reflecting that such sites are both disturbed and rich in nutrients^34^. Contrary to that, plots on crests are less diverse but host the largest share of species considered important indicators of vegetation units typical for KNP^9^.

The significant interaction between bedrock and water availability shows that the response of both herbs and shrubs to water availability is specific for granites and basalts. The most apparent aspect of this difference is the remarkable dominance of grasses (*Bothriochloa radicans*, *Panicum coloratum*, *Themeda triandra*) on basaltic crests compared to those on granites.

### The importance of seasonal rivers: disturbance by herbivores and water availability

The effect of herbivores, elephants in particular, on vegetation has been thoroughly studied^49^, yet not in the perspective of the two factors addressed in our study or directly exploring the role of seasonal rivers. With high population densities, elephant herds influence the savanna ecosystem not only by consuming large amounts of plant tissue (mainly leaves) but also by damaging or uprooting grown trees^18,50^, hence changing woody savanna into more open grass dominated states^51,52^. While some plant species cope with these disturbances well, other important tree canopy species regenerate poorly^53^. Other species regenerate more easily but cannot cope with bark-stripping and thus continued elephant use keeps them from attaining the size they would reach in the absence of elephants^54^. Bark-stripping also makes the trees more vulnerable to fungal infection, insect attacks, and fire^55,56^. Elephants also affect trees by pulling out seedlings or eating their apical meristems, disabling them to grow to a size at which they can survive fires^28,57^. The impact of other large herbivores can be even stronger. For example, Scogings et al.^58^ reported that species richness and density of woody plants increased five years after exclusion of all large herbivores, but not after the exclusion of elephants alone (see also Barnes^57^). The joint impact of all herbivore species on vegetation influenced not only species richness but also vegetation structure, as documented by Asner et al.^19^ who found that 3-D structure of woody vegetation differed significantly between areas protected from and accessible to herbivores, with up to 11-fold greater woody canopy cover in the former areas.

The vegetation on crests, where water availability is most limiting of the three habitats we sampled, hosts a lower diversity of herbs than seasonal or perennial rivers. Shrubs represent a life history that is most exposed to herbivores’ impacts, and as such, they followed a different pattern in our study—they reached ~ 26–27% greater species richness near seasonal rivers than in the other two habitats. While the difference in shrub species richness between seasonal rivers vs. perennial rivers and crests was only marginally significant (which can be attributed to a low sample size given by the field research logistics), it seems to hold for both bedrock types. Seasonal river plots on granites harboured on average 31% more shrub species than perennial rivers and 24% more than crests, with corresponding figures on basalt being 18% and 32%, respectively (Fig. 5D).

This finding supports our prediction; we hypothesized that shrubs near perennial rivers might be damaged by animals or riparian disturbances^59^, while shrubs on crests may suffer from low water availability, mechanical root damage and the influence of fire^60^. Therefore, seasonal rivers where the relatively low disturbance combined with a low stress from the lack of water seem to be the habitat that supports the greatest richness and diversity of shrubs, while the total shrub cover remains at a comparable level to the other two habitats (~ 35%). This conclusion is further supported by the fact that seasonal rivers harboured the greatest number of woody species that occurred only in this habitat (Supplementary Material File 2).

Rivers are an important factor driving elephant distribution and densities, as these animals generally concentrate closer to major rivers^61^. Smit and Ferreira^29^ further suggested that based on aerial census data collected annually during the mid-dry season of 1985–2007 across KNP, rivers of different sizes can be used as a proxy for elephant densities in the dry period of the year. They showed that since 1999 when water provision in KNP was reduced and culling ceased, elephant densities at seasonal rivers (both large and intermediate) were lower than at perennial rivers and higher than at sites far from rivers. Assuming a correlation between elephant density and elephant impact, these authors report a disproportionally greater increase in impact around large rather than small rivers, including seasonal, and crests^29^. Together with our data on occurrences of all herbivores recorded by camera traps, these findings support our hypothesis of the greatest shrub species richness at seasonal rivers due to relatively lower disturbance, interacting with water availability gradient.

The signal we observed, pointing to the role of seasonal rivers as supporting high species richness of shrubs, a key plant life form of the savanna ecosystem, is consistent across landsystems and supported by data on the herbivores’ habitat affinities. From the management perspective, the role of seasonal rivers in maintaining high diversity of woody vegetation should be taken into account when predicting the risk of local extirpation of specific woody species^29,62^, especially in light of increasing elephant densities closer to rivers^61^.

### Too hot to handle: the effect of large-scale factors

The only strong signal from the large-scale analyses was related to temperature. The variation in Enhanced Vegetation Index, representing the fluctuation in vegetation phenology over time and space^63^, and distance from the gravel roads, which can be interpreted as opportunities for rapid propagule dispersal, were also identified as important in determining plant species richness, and cover. However, their effects were only marginally significant and not consistent, depending on whether or not the spatial effects were included in the models. Other variables such as the number and intensity of fires, rainfall characteristics, or the presence of surface waters did not show significant effects in our models. This does not mean they are not important, rather the opposite is true as evidenced by decades of botanical research in South-African savannas^5,11,12^. Within our system, however, the results strongly suggest that habitat, bedrock, and herbivores are the key determinants of local plants species richness, cover, and community composition. We argue that large-scale factors, such as temperature, further influence the effects of these factors thereby explaining part of the residual variation. The non-significant effects of some park-wide large-scale factors can be further related to complex spatiotemporal interactions. For example, the possible effects of the presence of surface water, related to soil moisture as a critical factor for plants, could be overwhelmed by the water availability in nearby rivers. Similarly, the effect of rainfall and possibly fires will also manifest in the variation in vegetation productivity. Or, the effect of fire on plant richness could be masked by the direct effect of bedrock—this would be indicated by the number of fires on granites in our system being marginally significantly greater than on basalts (*p* = 0.099). In addition, we faced a statistical limitation associated with the sampling design—the power of all tests was limited due to rather low number of independent replicates (20 triplets, 60 plots altogether). On the other hand, the plots were spread across the whole area of KNP and therefore can be assumed to be representative for the effects of large-scale predictors, such as climate or fire regime.

The temperature in KNP is negatively related to the species richness of all target groups of plants, i.e., shrubs, herbs, and all species. Here, it needs to be emphasized that our models allowed us to identify the pure effects of temperature, not confounded by other variables and geography of KNP. This shows that high temperatures are associated with low plant diversity in a situation when other important drivers (bedrock, water availability, geography) are constant, suggesting that it is the heat stress what limits the plant diversity. Indeed, plant diversity in warm regions with a limited water availability decreases with increasing temperature^64,65^. This pattern, however, is only poorly documented in savannas. For example, Knight et al.^66^ found a strong negative relationship between annual solar radiation and plant richness in southern-African savannas.

As expected, the effect of the mopane cover on plant characteristics was heavily confounded with the north–south gradient in KNP. However, the partial effects of the mopane cover (after accounting for the north–south gradient) were not significant and were featured by low portions of explained variability. On the contrary, the effects of the north–south gradient were significant even after accounting for mopane. Although the south of KNP hosts richer flora, and mopane dominates the vegetation only in the north, we suggest that the high cover of mopane does not reduce the floristic richness in the north of the park, at least not so dramatically as previously thought^33^. Besides the statistical analyses we present here, we have also recorded several plots with a high cover of mopane rich in herb flora. For example, plots within a triplet near Phalaborwa (PHA2) hosted 75, 88 and 87 herb species at perennial river, seasonal river and on crest, respectively, and the covers of mopane at these plots ranges between ~ 40–70%.

### Species indicating the representativeness of savanna vegetation

Interestingly, crests showed the highest proportion of herb and shrub species considered as indicators of typical vegetation (as defined in Mucina and Rutherford^9^), contrary to plots at perennial rivers, which harboured the least indicator species typical for those habitats. This result shows that even though the plots around perennial rivers were species-rich, a part of their diversity was due to the occurrence of widespread weeds (*Acalypha indica*, *Amaranthus praetermissus*, *Pupalia lappacea*) or invasive aliens (*Acanthospermum hispidum*, *Alternanthera pungens*, *Gomphrena celosioides*^34^). The affinity of weedy species to perennial rivers is also the reason why this habitat hosted the highest numbers of forbs occurring only there.

To conclude, plant species richness, diversity, composition, and cover all respond to bedrock and water availability in South African savannas, as well as to their interactions. For the woody life histories, the effects of these factors are fine-tuned by herbivory. The plant community characteristics, however, differ in their response to the main factors. For example, crests exhibit the lowest species richness and diversity, but the proportion of ‘important species’ (sensu Mucina and Rutherford^9^) indicating typical, well-developed vegetation units is the highest of all habitats. Similarly, herbs, including grasses, reach the highest cover on basaltic crests, suggesting that their lowest diversity in this habitat may be associated with the high biomass of a few dominants.

These results indicate that the typical vegetation of different habitats is associated with manifold characteristics. The crests, exposed to relatively low pressure from grazing but stressed by the lack of water, are important from the conservation perspective because of the presence of typical, sometimes rare savanna species, and so are seasonal rivers whose shrub richness is increased and maintained by the combination of low levels of disturbance and position in the middle of the water availability gradient.

## Methods

### Study area

Kruger National Park (KNP) is one of the largest nature reserves in South Africa and one of the world’s oldest national parks (established in 1926). It is located in the north-eastern part of the country, covering an area of 19,485 km^2^, and stretching ~ 450 km in a north–south direction. The majority of the park is in a subtropical climate, with the Tropic of Capricorn crossing the park in its northern part. Five perennial rivers run through the park, mostly in the west–east direction, including Sabie, Olifants, Crocodile, Letaba, and Luvuvhu^67^. The park is divided into 11 landscape systems, of which four (Skukuza—20% of the park area, Satara—14%, Letaba—18%, and Phalaborwa—26%) dominate 78% of the park^68^. These landsystems reflect the environmental heterogeneity generated by geological conditions (granitoid bedrock in the western vs. volcanic, mainly basalt and gabbro, in the eastern part), altitude (140–780 m a.s.l.), climate (450–750 mm of annual precipitation) and character of vegetation (dominant woody species, the proportional representation of woody cover vs. open grassland in KNP)^37,69,70^.

The beginning of botanical research in KNP dates back to the 1930s, when the first list of over 300 plant species was compiled^71^. Almost 30 years later, van der Schijff^72,73^ reported 1800 species of flora in the KNP and discussed their phytogeographical relationships with neighbouring regions. The last published work inventorying KNP’s flora list 1903 species, including over 400 tree and shrub species, and over 220 grasses^35^. In terms of vegetation studies, plant communities in KNP were described by using a phytosociological approach. Coetzee^74^ provided a detailed classification of plant communities in the central KNP, and Gertenbach^75^ defined 35 landscape types classified based on vegetation composition and other factors. In the 2000s, the vegetation of the whole park was classified into phytosociological units within the Vegetation of South Africa project^9^. More recent works classified and mapped vegetation within enclosures^76,77^, studied the effect of exclusion of large herbivores on vegetation composition^58,78^, evaluated the effect of fire on vegetation dynamics and structure^26,79^, related the species composition and productivity of vegetation to the availability of surface water^27,80,81^, focused on woody species^58,78–80^, explored bottom-up effects of water availability and substrate^17^, or assessed plant invasions in KNP^34,82,83^.

### Sampling and field data

MOSAIK’s primary objective is to sample plant and animal (mammal, bird, and insect) communities in habitats across the four main landsystems in KNP. To account for differences in water availability, we used plots arranged in spatially defined triplets, each comprising three habitats: (i) near a perennial river or another permanent water source, such as artificial water points or dams, (ii) near a seasonal river with a lack of water during dry periods, and (iii) on dry crests, at least 5 km from any source of water (Fig. 1). Each triplet was located on either granitic (Skukuza and Phalaborwa landsystems) or basaltic (Satara and Letaba landsystems) bedrock^68^ (Fig. 1). Therefore, we captured the effects of two factors: type of bedrock, defined at the level of triplets, and habitat representing water availability, defined at the level of individual plots within each triplet. The plots within each triplet were selected within a distance of ~ 7–13 km between them. In total, we sampled vegetation in 60 plots, 50 × 50 m in size, each bedrock represented by 30 plots, and each habitat by 20 plots across the entire KNP (Fig. 2). The plots were selected with a focus on shrubby savanna; those by perennial rivers were located behind the riparian gallery forest, and although some trees occasionally occurred in plant inventories, they were not included in analyses here.

From the vegetation perspective, there are 19 vegetation types in KNP based on phytosociological classification^9^, of which 13 were covered by our plots; most represented were SVl3 Granite Lowveld (13 plots), SVmp4 Mopane Basalt Shrubland (12 plots), SVl5 Tshokwane-Hlane Basalt Lowveld (10 plots), and SVmp5 Tsende Mopaneveld (9 plots).

Plants were sampled during two rainy seasons (when the vegetation is in optimal phenological stage allowing sampling), 16 January to 4 February 2019 and 17 January to 3 February 2020; we sampled 33 and 27 plots, respectively, stratified so as to proportionally represent all landsystems and habitats in each sampling period. The January rainfall for KNP in 2019 and 2020 was 88 mm and 97 mm, respectively (averaged across all KNP stations). All vascular plant species (for simplicity, intraspecific taxa such as subspecies were included under the term ‘species’) were recorded in each 2500 m^2^ plot. Their abundance was visually estimated using the Braun-Blanquet cover-abundance seven-grade scale^84^. To quantify the occurrence of species in plots, the Braun-Blanquet scores were transformed to percentage values as follows: 5 = 87.5%, 4 = 62.5%, 3 = 37.5%, 2 = 15%, 1 = 2.5%, +  = 1.0%, r = 0.02%^85^. Abundance within the herb, shrub, and tree layers was estimated separately. The time spent by three botanists to sample a plot ranged from 1 to 7 h, with an average of 2:15 ± 1:01 h (mean ± S.D.); the same three botanists sampled all sites.

Plant species were classified according to life form (herbs, shrubs) and in each plot, the following characteristics were calculated separately for each of these life forms and used as response variables in statistical analyses: (i) species richness expressed as the total number of species in a plot; (ii) species diversity expressed as the Shannon index H’, calculated using species percentage cover as importance values^86^; (iii) total cover of the herb and shrub communities (%) estimated in the field^84^. To assess the extent to which the vegetation in plots represents typical natural vegetation as defined for South Africa, we identified species that are labelled as ‘important taxa’ for the respective vegetation units and indicate their phytosociological assignment^9^. Species labelled as such in any of the 13 vegetation units captured by our sampling are further termed as ‘indicator species,’ and (iv) their proportion was used as an additional characteristic analysed.

The nomenclature of vascular plants follows^87–90^.

To estimate the numbers of herbivores in the three habitats and relate the extent of disturbance to vegetation with botanical data, we used camera traps located in the same plots (n = 60), one in each (Bushnell Essential E3 Camera Trap with low glow IR flash). The cameras were serviced once in ~ 3 months, starting in June 2018. The monitoring was part of the MOSAIK project, aimed at recording the diversity and activity of mammals. Here we use animal records over 140 days, including both the dry season (August–October 2018) and the rainy season (December 2018–February 2019). For each species, we calculated the number of animal records taken by the camera in a given site. The overall herbivore load per plot was then expressed as the sum of all the herbivore-species’ records, i.e. reflecting their abundances. We considered large herbivores and megaherbivores, both browsers, grazers and mixed feeders, where an impact on vegetation is expected (18 species in total): buffalo, bushbuck, common duiker, elephant, giraffe, grysbok, hippo, impala, kudu, nyala, black rhino, white rhino, sable antelope, steenbok, tsessebe, waterbuck, wildebeest, and zebra.

### Large-scale variables

To analyse the effect of large-scale factors on plant species richness and cover, we collected large-scale variables related to the fire history (number of fires and their frequency), vegetation productivity (Enhanced Vegetation Index, EVI), rainfall (sum and mean), temperature (mean, minimum, maximum) and surface-water occurrence density (sum, mean). All these variables were measured in a 4-km^2^ grid cell surrounding the sample plot and over the long-term (2000–2019). For all variables, we also included the measure of their variation over time (see Supplementary Table 1 for detailed description and data sources). In addition, we also measured the distance of the plot from rivers, roads, restcamps, and Kruger boundary, to account for opportunities of propagule dispersal, both naturally and by human action (Supplementary Table 1).

### Data analysis

The differences in herb and shrub species richness, Shannon diversity H’, herb and shrub total covers, proportions of indicator species, and herbivore numbers were analysed using LMM regression models within the *nlme* package of R software^91^. The identity of a triplet was arranged as a random factor (grouping variable), and the spatial distances between individual triplets were modelled as a continuous function, based on the GPS coordinates. As a result, the univariate LMM model on species richness, diversity, or total cover was: lme(response variable ~ bedrock*habitat, random =  ~ 1|triplet identity, cor = corGaus(form =  ~ gpsn + gpse)). The differences between habitats (perennial rivers, seasonal rivers, crests), if significant, were subsequently tested by the Tukey *posthoc* pairwise comparison of estimated marginal means, using the *emmeans* package in R^92^. The data on total covers and proportions of indicator species were arcsine transformed. The data on species richness and Shannon diversity H’ were square-rooted if Shapiro–Wilk test revealed significant deviations from normality.

The differences in species composition between the two bedrock types (basalt vs. granite) and the three habitats reflecting water availability (perennial rivers, seasonal rivers, crests) were tested using multivariate ordination methods and Monte-Carlo permutation tests in Canoco 5 software^93^. A hierarchical split-plot arrangement was used to account for the pseudoreplication structure in the data: individual triplets were defined as whole plots and the permutations at the whole-plot level tested the main effect of bedrock. In contrast, permutations within the whole plots tested the main effect of habitat (water availability), a factor defined at the split-plot level. Finally, a model testing the bedrock × habitat interaction was created by permuting the whole-plots as well as split-plots.

The spatial effects, given by the location of individual triplets (whole-plots) were accounted for by the method of PCNM (Principal Coordinates of Neighbour Matrices^9^), which uses the Euclidian spatial distances to create a matrix of spatial vectors, independently at different scales^94,95^. The scores of the first three spatial PCoA vectors were then used as covariables in the ordination models that accounted for the spatial effects.

To explore how the large-scale factors are correlated in affecting plant species richness and cover, we used the direct gradient ordination analyses (RDA). Total plant richness, herb and shrub species richness, and covers of herbs, shrubs and grasses (in %) were response variables; the large-scale factors listed in Supplementary Table 1 were predictors (Table 1). Here, we also analysed the total vascular plant species richness, which is traditionally the main variable of interest in large-scale analyses, i.e. not only shrub and herb as in analyses of local factors where we were more interested in vegetation structure defined by vegetation layers; for the same reasons the Shannon diversity H’ was only tested in local-scale analyses, not in the large-scale one. The plots were permuted in a split-plot scheme to account for the arrangement of individual plots into triplets. Individual triplets were defined as whole plots, while the split-plots were represented by individual vegetation plots within triplets. The manual forward selection of environmental variables was used to identify the large-scale predictors with the strongest explanatory powers. Further, the bedrock (basalt, granite) and habitat (perennial river, seasonal river, crest) were set as covariables because we were interested in the variability beyond that explained by these local factors. The method of PCNM was used to account for spatial effects, beyond those given by the arrangement of individual plots into triplets. As a result, the first three principal coordinates (PCO1, PCO2, PCO3) were included in the ordination models as covariables representing spatial effects.

To test the significance of the relationships between large-scale factors and plant species richness/cover, indicated by the ordination methods (Fig. 8), we used linear regression models. First, the effects of principal coordinates (PCO1, PCO2, PCO3) on a particular richness/cover measure were tested by a linear regression model. Then, we ran an LMM model with bedrock, habitat, bedrock × habitat interaction, principal coordinates with significant effects, triplet as a random effect, and richness/cover as a response variable. Finally, the effects of large-scale factors were tested in a regression model, with residuals from the corresponding LMM model as a response variable. By doing this, we accounted for (i) the effect of bedrock, habitat and their interaction, (ii) the effect of spatial autocorrelation caused by the arrangements of individual plots in triplets, and (iii) the autocorrelation given by the distribution of individual plots as well as triplets in the whole target area of KNP.

The effect of the *Colophospermum mopane* cover (estimated in the sampling plots in the field) on species richness (total, herbs, shrubs) was tested by using a linear regression model. Variance partitioning was applied to separate the effects of the mopane cover from those of the north–south gradient. Further, the partial effects of mopane and the north–south gradient were tested after accounting for the effects of bedrock (granite, basalt), habitat (perennial river, seasonal river, crest), and their interaction, similarly to the effects of the large-scale predictors. The effects of the mopane cover and north–south gradient were tested on residuals from the LMM model with bedrock and habitat as predictors, and triplet as a random effect.

## Supplementary Information

Supplementary Information.
