# Supplementary material for: Water availability, bedrock, disturbance by herbivores, and climate determine plant diversity in South-African savanna
Source: Sci Rep. 2022 Jan 10;12:338. doi: 10.1038/s41598-021-02870-3 (PMC8748544; doi:10.1038/s41598-021-02870-3)
Supplement: Supplementary file 1 — Supplementary Information. [file 41598_2021_2870_MOESM1_ESM.docx]

**Supplementary Table 1.** Overview of variables used to analyse the effect of macroecological factors on plants species richness and cover in the studied plots. All variables are comprised of 4-km^2^ grid cells (2 × 2 km) covering the whole park. The spatial intersection of grid-cells and centre points of each 50 × 50 m sample plot were derived using extract function in raster package (Hijmans 2021). Descriptions and data source references are given below for each variable. “Long-term” represents 20 years, from 2000 to 2019. The distance-related variables were derived from different vector layers (spatial-points, -lines or –polygons) using gridDistance function in raster package (Hijmans 2021).

| **Column** | **Name** | **Description** | **Description/source** |
| --- | --- | --- | --- |
| fireSum | Fire frequency | Total number of fires per 4 km^2^ grid cell from 2000 to 2019; that is, the sum of the number of times each pixel was classified as burnt over a 20-year period. | Chuvieco et al. 2018; https://developers.google.com/earth-engine/datasets/catalog/ESA_CCI_FireCCI_5_1 |
| fireMean | Mean number of fires | Pixel-wise mean of the number of fires per 4 km^2^ grid cell recorded over the long-term. | Chuvieco et al. 2018; https://developers.google.com/earth-engine/datasets/catalog/ESA_CCI_FireCCI_5_1 |
| fireSD | StdDev of number of fires | Long-term standard deviation of the number of fires per 4 km^2^ grid cell, as described above. | Chuvieco et al. 2018; https://developers.google.com/earth-engine/datasets/catalog/ESA_CCI_FireCCI_5_1 |
| eviSum | Sum EVI | Long-term sum of Enhanced Vegetation Index (EVI) per 4 km^2^ grid cell. | MOD13Q1.006 Terra Vegetation Indices 16-Day Global 250m (mean); Didan et al. 2015 |
| eviMean | Mean EVI | Long-term mean of Enhanced Vegetation Index (EVI) per 4 km^2^ grid cell. | MOD13Q1.006 Terra Vegetation Indices 16-Day Global 250m (mean); Didan et al. 2015 |
| eviSD | StdDev EVI | Long-term standard deviation of Enhanced Vegetation Index (EVI) per 4 km^2^ grid cell. | MOD13Q1.006 Terra Vegetation Indices 16-Day Global 250m (mean); Didan et al. 2015 |
| rainSum | Sum rainfall | Long-term sum of all rainfall per 4 km^2^ grid cell. | CHIRPS Pentad: Climate Hazards Group InfraRed Precipitation with Station Data (version 2.0 final) (sum); Funk et al. 2015 |
| rainMean | Mean rainfall | Long-term mean of all rainfall per 4 km^2^ grid cell. | CHIRPS Pentad: Climate Hazards Group InfraRed Precipitation with Station Data (version 2.0 final) (sum); Funk et al. 2015 |
| rainSD | StdDev of rainfall | Long-term standard deviation of all rainfall per 4 km^2^ grid cell. | CHIRPS Pentad: Climate Hazards Group InfraRed Precipitation with Station Data (version 2.0 final) (sum); Funk et al. 2015 |
| tempMean | Mean temperature | Long-term mean of temperature (°C) per 4 km^2^ grid cell. | MOD11A2.006 Terra Land Surface Temperature and Emissivity 8-Day Global 1 km (mean); Wan et al. 2015 |
| tempSD | StdDev of temperature | Long-term standard deviation of temperature (°C) per 4 km^2^ grid cell. | MOD11A2.006 Terra Land Surface Temperature and Emissivity 8-Day Global 1 km (mean); Wan et al. 2015 |
| tempMin | Minimum temperature | Long-term mean of minimum temperature (°C) per 4 km^2^ grid cell. | MOD11A2.006 Terra Land Surface Temperature and Emissivity 8-Day Global 1 km (mean); Wan et al. 2015 |
| tempMax | Maximum temperature | Long-term mean of maximum temperature (°C) per 4 km^2^ grid cell. | MOD11A2.006 Terra Land Surface Temperature and Emissivity 8-Day Global 1 km (mean); Wan et al. 2015 |
| waterSum | Sum surface water occurrence density | Long-term sum of surface water occurrence, expressed as kernel density estimates (KDE) per 4 km^2^ grid cell. | Annual density of water extent, i.e. weighed by % re-occurrence of water each year; Pekel et al. 2016 |
| waterMean | Mean surface water occurrence density | Long-term mean of surface water occurrence, expressed as kernel density estimates (KDE) per 4 km^2^ grid cell. | Annual density of water extent, i.e. weighed by % re-occurrence of water each year; Pekel et al. 2016 |
| waterSD | StdDev surface water occurrence density | Long-term standard deviation surface water occurrence, expressed as kernel density estimates (KDE) per 4 km^2^ grid cell. | Annual density of water extent, i.e. weighed by % re-occurrence of water each year; Pekel et al. 2016 |
| distBnd | Distance to Kruger boundary | Distance to Kruger boundary (mean of surrounding 4 km^2^ grid cell). | SANParks 2021 |
| distCamp | Distance to all restcamps | Distance to all restcamps (mean of surrounding 4 km^2^ grid cell). | SANParks 2021 |
| distTar | Distance to all tarred roads | Distance to all tarred roads (mean of surrounding 4 km^2^ grid cell). | SANParks 2021 |
| distDirt | Distance to all gravel roads | Distance to all gravel roads (mean of surrounding 4 km^2^ grid cell). | SANParks 2021 |
| distRiv | Distance to all major rivers | Distance to all major rivers (mean of surrounding 4 km^2^ grid cell). | SANParks 2021 |
| distStrm | Distance to all rivers and streams | Distance to all rivers and streams (mean of surrounding 4 km^2^ grid cell). | SANParks 2021 |

References

Chuvieco, E., Pettinari, M. L., Lizundia-Loiola, J., Storm, T., Padilla Parellada, M. (2018) ESA Fire Climate Change Initiative (Fire_cci): MODIS Fire_cci Burned Area Pixel product, version 5.1. Centre for Environmental Data Analysis, 01 November 2018. <https://doi.org/10.5285/58f00d8814064b79a0c49662ad3af537>.

Didan, K. (2015) MOD13Q1 MODIS/Terra Vegetation Indices 16-Day L3 Global 250m SIN Grid V006 [Data set]. NASA EOSDIS Land Processes DAAC. Accessed 2021-06-08 from <https://doi.org/10.5067/MODIS/MOD13Q1.006>

Funk, C., Peterson, P., Landsfeld, M., Pedreros, D., Verdin, J., Shukla, S., Husak, G., Rowland, J., Harrison, L., Hoell, A, Michaelsen, J. (2015) The climate hazards infrared precipitation with stations—a new environmental record for monitoring extremes. Scientific Data 2, 150066. doi:10.1038/sdata.2015.66 2015

Hijmans, R. J. (2021) raster: Geographic Data Analysis and Modeling. R package version 3.4-10. https://CRAN.R-project.org/package=raster

Pekel, J.-F., Cottam, A., Gorelick, N., Belward, A. S. (2016) High-resolution mapping of global surface water and its long-term changes. Nature 540, 418-422. doi:10.1038/nature20584)

SANParks (2021) Geographic Information Systems unpublished spatial dataset. South African National Parks (SANParks). url: http://dataknp.sanparks.org/.

Wan, Z., Hook, S., Hulley, G. (2015) <i>MOD11A1 MODIS/Terra Land Surface Temperature/Emissivity Daily L3 Global 1km SIN Grid V006</i> [Data set]. NASA EOSDIS Land Processes DAAC. Accessed 2021-06-08 from <https://doi.org/10.5067/MODIS/MOD11A1.006>

| A. Forbs | B. Grasses |
| --- | --- |
| 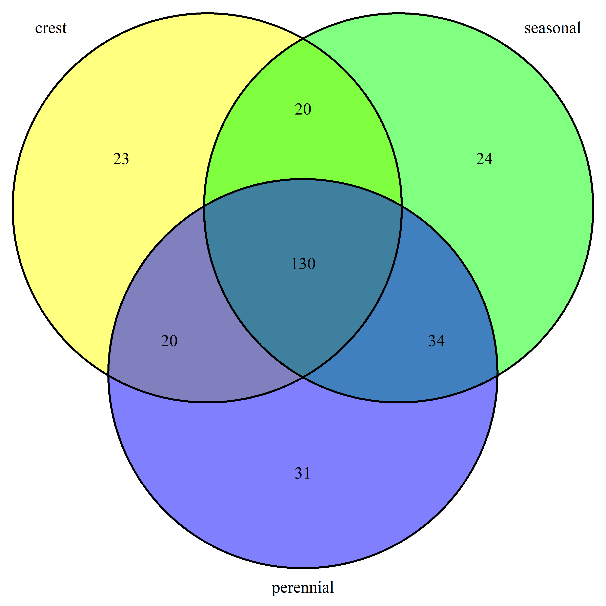 | 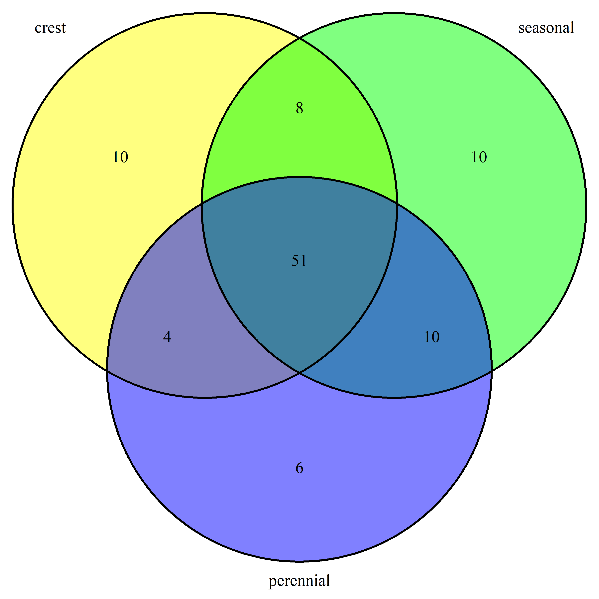 |
| C. Woody | D. Bedrock: all |
| 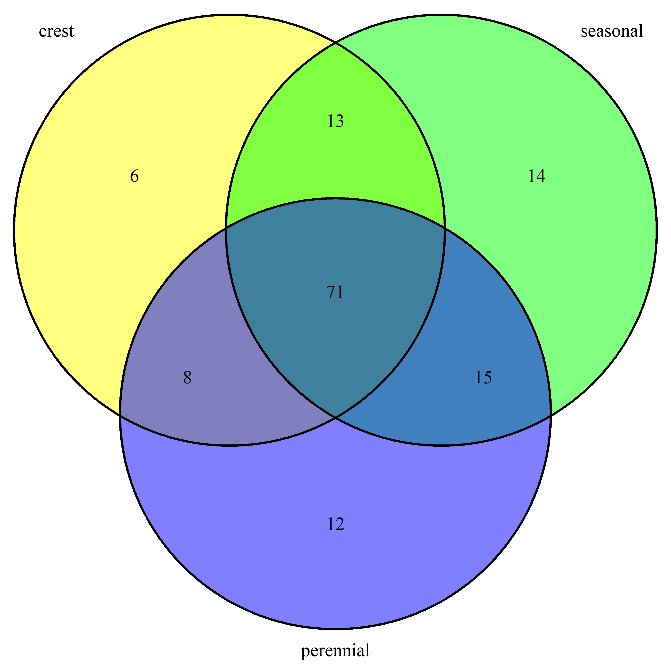 | 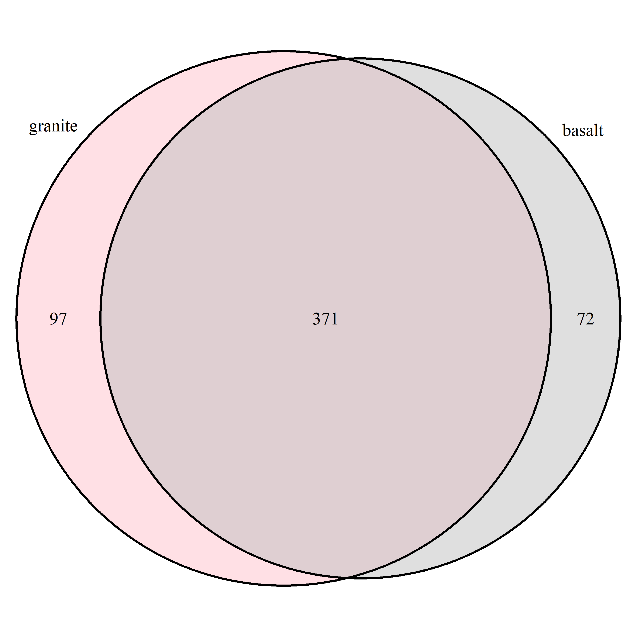 |

Supplementary Fig. 1. Venn diagram showing the sharing of species, separately for life histories (forbs, grasses, woody) by habitats (A–C) and by bedrock (for all species, D) in the Kruger National Park.
